# Supplementary material for: Endothelial and Cardiovascular Effects of Naringin: A Systematic Review
Source: Nutrients. 2025 Aug 17;17(16):2658. doi: 10.3390/nu17162658 (PMC12389103; doi:10.3390/nu17162658)

Supplementary Materials

Systematic Review

Endothelial and Cardiovascular Effects of Naringin: A Systematic Review

Jose A Adams , Arkady Uryash , Alfredo Mijares , Jose Miguel Eltit and Jose R Lopez

Table S1: Cell Studies from Jan 2000 to June 2025 on the Effects of Naringin with Key Outcomes.

Reference and Year	Cell Type	Disease Model	Dosage of Naringin Used	Duration of Treatment	KEY Outcomes
Ajay et al. (2003)	Rat thoracic aorta rings (ex vivo)	Phenylephrine/KCl-induced contraction	1–100 µM	Acute exposure	Induced vasorelaxation; NO/prostaglandin involvement; inhibited Ca^{2+} influx
Balestrieri et al. (2003)	Endothelial cells	H_2O_2-induced oxidative stress	Pre-treatment (concentration not specified)	Acute exposure	Activated transacetylase activity; enhanced acyl-PAF synthesis; antioxidant effect
Kim et al. (2003)	Rat vascular smooth muscle cells (VSMCs)	Lysophosphatidylcholine-induced proliferation	0, 10, 100 µM	24 h	Suppressed lysophosphatidylcholine-induced VSMC proliferation
Saponara et al. (2006)	Rat tail artery myocytes	Electrophysiological evaluation (no damage model)		Acute exposure	Opened BKCa channels; induced vasodilation via K^+ efflux
Lee et al. (2008)	Rat vascular smooth muscle cells (VSMCs)	Proliferation and cell cycle dysregulation	Not explicitly stated	Not explicitly stated	Induced p21WAF1-mediated G1 arrest via Ras/Raf/ERK pathway; suppressed cyclin/CDK expression
Lee et al. (2009)	Vascular smooth muscle cells (VSMC)	TNF-α-induced migration and inflammation	10–25 µM	Pre-treatment	Inhibited MMP-9, IL-6/8, AKT; suppressed migration/invasion via PI3K/AKT/mTOR inhibition

Reference and Year	Cell Type	Disease Model	Dosage of Naringin Used	Duration of Treatment	KEY Outcomes
Kim et al. (2011)	Human umbilical vein endothelial cells (HUVECs)	High-glucose-induced ICAM-1 expression	50 µM	6 h pre-treatment	Reduced ICAM-1 via p38 MAPK inhibition
Rizza et al. (2011)	Bovine aortic endothelial cells (BAEC)	TNF-α-induced endothelial inflammation	10 µM hesperetin (metabolite of naringin)	5 h	Stimulated eNOS, Akt, AMPK phosphorylation; reduced VCAM-1 and monocyte adhesion; NO production increased
Chen et al. (2013)	H9c2 cardiomyoblasts	High-glucose (35 mM)-induced injury	80 µM	2 h pre-treatment + 24 h HG exposure	Inhibited ROS/MAPK (p38, ERK, JNK); improved viability, reduced apoptosis and oxidative stress
Huang et al. (2013)	H9c2 cardiomyocytes	High-glucose-induced apoptosis	80 µM	2 h pre-treatment + 24 h HG exposure	Reduced caspase-3/8/9, p38/p53, Bax/Bak; increased Bcl-2 and MMP
Chen et al. (2014)	H9c2 cardiomyoblasts	High-glucose injury via leptin-induced p38 MAPK	80 µM	2 h pre-treatment + 24 h HG exposure	Suppressed leptin/p38 MAPK activation; reduced ROS and apoptosis
Gutiérrez-Venegas et al. (2014)	H9c2 cardiomyocytes	LTA-stimulated inflammation	10 µM	Pre-treatment before LTA	Reduced iNOS, NF-κB, ERK, JNK, p38; anti-inflammatory effect
Li et al. (2014)	HUVECs	TNF-α-induced inflammation	Not explicitly stated (micromolar range)	Pre-treatment	Reduced ROS, Nox4, ICAM-1, VCAM-1; inhibited NF-κB and PI3K/Akt

Chen et al. (2015)	H9c2 cardiomyoblasts	Anoxia/reoxygenation-induced apoptosis	1–40 μM (optimal dose 10–20 μM)	6 h pre-treatment	Reduced ROS and apoptosis via Nrf2 activation; increased HO-1, GCLC, SOD, CAT, GPx
**Reference and Year**	**Cell Type**	**Disease Model**	**Dosage of Naringin Used**	**Duration of Treatment**	**KEY Outcomes**
Bi et al. (2016)	HUVECs	LPS-induced inflammation and apoptosis	Not explicitly stated	Pre-treatment	Reduced ROS, cytokines, MAPK phosphorylation; improved cell survival and mitochondrial integrity
You et al. (2016)	H9c2 cardiomyocytes	Hyperglycemia-induced injury	Not explicitly stated	24 h high glucose exposure with pre-treatment	Reduced apoptosis, ROS, NF-κB; upregulated KATP channels; improved mitochondrial function
Jian et al. (2017)	H9c2 cardiomyocytes	Doxorubicin-induced cardiotoxicity	1 μM	150 min pre-treatment	Reduced ROS and p-p38; improved viability and mitochondrial protection
Li et al. (2017)	HUVECs	High glucose-induced endothelial injury	Not explicitly stated; treatment group for 5 days	5 days	Reduced ROS, downregulated CX3CL1, restored NO and OCR, improved mitochondrial function
Wang et al. (2020)	HUVECs	HG/HF-induced endothelial dysfunction	86 μM	Not explicitly stated	Inhibited autophagy via PI3K-Akt-mTOR; restored function in stressed endothelial cells
Li et al. (2021)	H9c2 cardiomyocytes	CoCl2-induced chemical hypoxia	Not explicitly stated; pre-treatment protocol	Pre-treatment before CoCl2 exposure	Promoted autophagic flux via HIF-1α/BNIP3 signaling; decreased

					apoptosis and cytotoxicity
Luo et al. (2021)	H9c2 cardiomyocytes	High glucose-induced injury and inflammation	NRG pre-treatment (	24 h HG exposure with pre-treatment	Attenuated injury and inflammation via leptin-JAK2/STAT3 pathway
**Reference and Year**	**Cell Type**	**Disease Model**	**Dosage of Naringin Used**	**Duration of Treatment**	**KEY Outcomes**
Xu et al. (2021)	H9c2 cardiomyocytes	H/R injury with Erastin-induced ferroptosis		Pre-treatment before H/R	Inhibited ferroptosis via Nrf2/System xc-/GPX4 axis; reduced infarction and oxidative injury
Uryash et al. (2021)	Cardiomyocytes from db/db mice	Type 2 diabetic cardiomyopathy	Oral administration (dose not stated)	4 weeks	Reduced [Ca2+]d, ROS, TNF-α, IL-6; restored KATP channel subunits and cardiomyocyte viability
Zhao & Zhao (2022)	HUVECs	TMAO-induced endothelial inflammation and oxidative stress	50, 75, 100 µM	2 h pre-treatment before TMAO	Reduced ROS and inflammation, preserved tight junction proteins, inhibited MAPK activation
Krga et al. (2022)	Peripheral blood mononuclear cells (PBMCs)	Baseline in postmenopausal women consuming grapefruit juice	340 ml GFJ daily (~210 mg naringenin glycosides)	6 months	Modulated inflammation- and vascular-related gene and miRNA expression; inverse correlation with PWV; pathways included PI3K-Akt, NF-κB, STAT3
Li et al. (2023)	Mouse ventricular cardiomyocytes	Electrophysiology/arrhythmia model	IC50 ~ 500 µM (concentration-dependent)	Acute exposure	Inhibited INa.L, ICa.L, IK, Ito; suppressed EADs, DADs, and arrhythmias

Mahadevaswamy et al. (2025)	H9c2 cardiomyocytes	Angiotensin II-induced cardiac hypertrophy	Not explicitly stated	Not specified	Reduced CA-II and NHE1 expression; inhibited cardiac hypertrophy via CA-II inhibition
Zhang et al. (2025)	H9c2 cardiomyocytes	Hypoxia/reoxygenation and ferroptosis	480 µM	Not explicitly stated	Improved mitochondrial function, reduced oxidative stress, inhibited cGAS-STING activation

Figure S1: Modified SYRCLE Based Risk of Bias for In-Vitro Studies

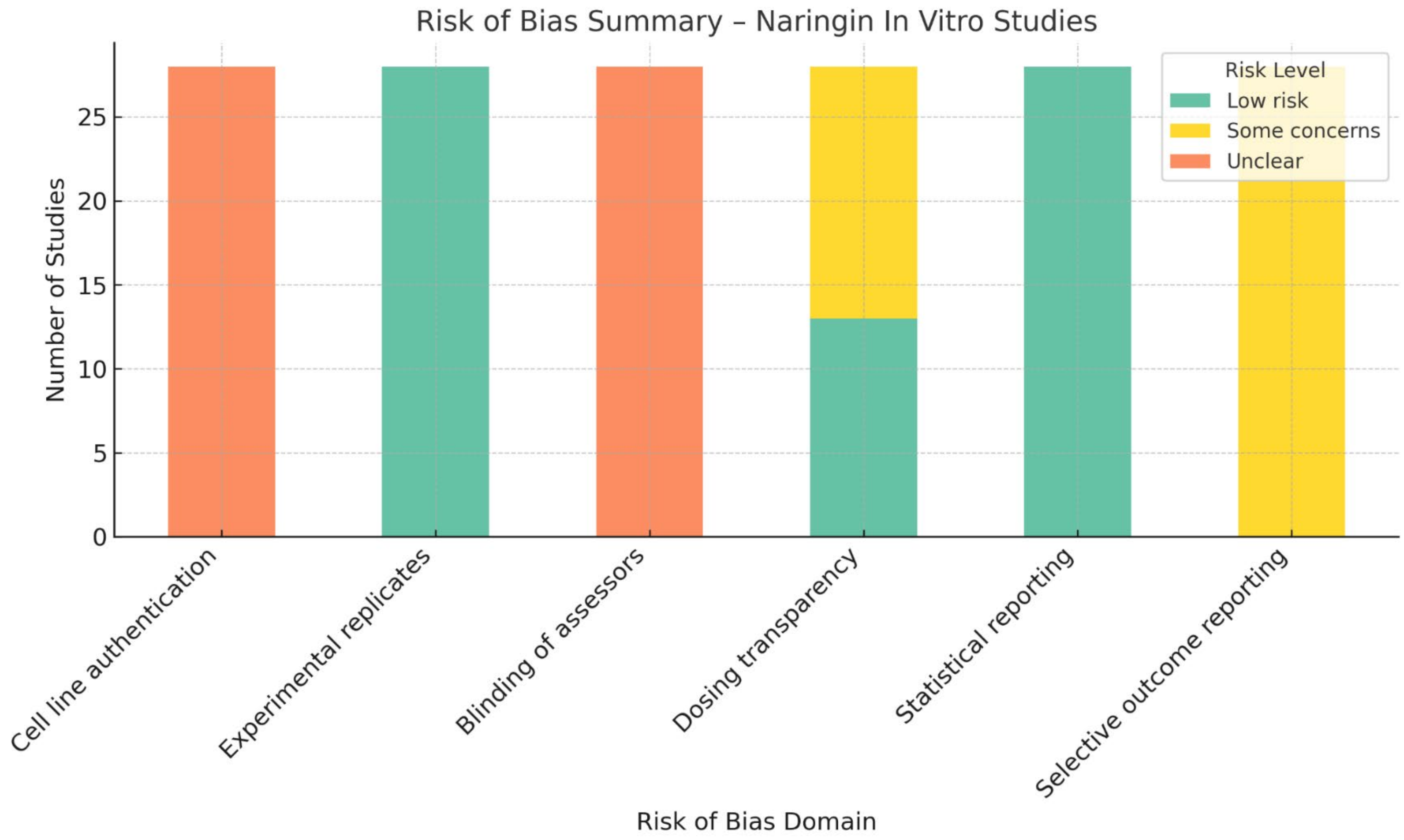

Table S2: Animal Studies from Jan 2000 to June 2025 on the Effects of Naringin on Cardiovascular Models with Key Outcomes

Reference and Year	Animal Model used	Dosage of Naringin Used	Duration of treatment	Key Outcomes
Lee et al. (2001)	High-cholesterol-fed New Zealand White rabbits	0.1% in diet	8 weeks	Reduced aortic fatty streaks, downregulated VCAM-1 and MCP-1
Choe et al. (2001)	Hypercholesterolemic rabbits	500 mg/kg/day	8 weeks	Reduced aortic fatty streaks and ICAM-1 expression
Jeon et al. (2004)	Rabbits fed high-cholesterol diet	0.05% in diet	8 weeks	Lowered LDL-C, increased HDL-C/Total-C, improved liver histopathology
Rajadurai & Prince (2006)	Wistar rats (ISO-induced myocardial infarction)	10, 20, and 40 mg/kg/day (oral)	56 days	Decreased lipids, improved lipid metabolism and reduced infarction damage
Rajadurai & Prince (2006)	Wistar rats (ISO-induced myocardial infarction)	10, 20, 40 mg/kg/day (oral)	56 days	Reduced lipid peroxidation and myocardial oxidative damage; histological protection
Sharma et al. (2011)	Wistar albino rats with HFD-STZ-induced type 2 diabetes	25, 50, and 100 mg/kg/day (oral)	28 days	Dose-dependent improvement in insulin resistance, β-cell function, dyslipidemia, hepatic steatosis, and kidney pathology; increased PPARγ, HSP-27, HSP-72; decreased NF-κB, CRP, TNF-α, IL-6; enhanced antioxidant enzymes and preserved islet morphology
Pu et al. (2012)	C57BL/6 mice (high-fat diet-induced metabolic syndrome)	Varied doses (not specified in snippet)	Several weeks (unspecified)	Improved insulin resistance, lipid metabolism, and oxidative stress via AMPK activation

Mahmoud et al. (2012)	High-fat fed/STZ-induced diabetic rats	50 mg/kg/day (oral)	4 weeks	Reduced glucose, HbA1c, MDA, TNF-α, IL-6; increased insulin and antioxidants
Pu et al. (2012)	C57BL/6 mice (high-fat diet model)	Not explicitly specified	Several weeks	Ameliorated metabolic syndrome via AMPK activation
**Reference and Year**	**Animal Model used**	**Dosage of Naringin Used**	**Duration of treatment**	**Key Outcomes**
Ikemura et al. (2012)	Stroke-prone spontaneously hypertensive rats (SHRSP)	Mixed in diet	4 weeks	Reduced blood pressure and cerebral thrombosis; improved NO bioavailability
Adebiyi et al. (2016)	Streptozotocin-induced type 1 diabetic rats (Sprague-Dawley)	50 mg/kg/day (oral)	56 days	Reduced myocardial fibrosis, oxidative stress, and PKC-β/p38 expression
Liu et al. (2016)	Murine endotoxemia model (LPS-induced)	Not specified; naringenin used up to 160 μM in vitro	Acute model	Suppressed inflammation via AMPK-ATF3-TLR4 signaling
Gil et al. (2016)	CLP-induced sepsis mice	200 mg/kg (oral)	Pre-treatment (acute study)	Reduced mortality and TNF-α, HMGB1 levels; involved HO-1/AMPK/p38/Nrf2 pathway
Malakul et al. (2018)	Sprague-Dawley rats (10% fructose in water)	100 mg/kg/day (oral)	4 weeks (last 4 weeks of 12-week fructose feeding)	Improved endothelial function via increased eNOS and NOx, reduced nitrotyrosine
Park et al. (2018)	Mice fed high-fructose diet	Not clearly stated	Acute and chronic time points used	Reduced cardiac hypertrophy via AMPK-mTOR and ATM-p53 signaling
Sun et al. (2019)	LPS-induced septic rats (Sprague-Dawley)	50 and 100 mg/kg/day (oral)	7 days pre-treatment	Improved myocardial strain, reduced cytokines and cardiac enzymes, modulated PI3K/AKT/NF-κB pathway
Yu et al. (2019)	Sprague-Dawley rats (MI/R injury model)	50 mg/kg/day (oral)	Before and after MI/R	Reduced oxidative and ER stress via cGMP-PKGIα signaling; improved heart function

Wang et al. (2019)	2-kidney, 1-clip hypertensive rats	200 mg/kg/day (oral)	10 weeks	Reduced renal damage and Ang II levels; normalized ACE/ACE2 and AT1R/AT2R ratios
Oyagbemi et al. (2020)	L-NAME-induced hypertensive rats	50 mg/kg/day (oral)	3 weeks	Normalized BP, improved oxidative stress and kidney injury markers
**Reference and Year**	**Animal Model used**	**Dosage of Naringin Used**	**Duration of treatment**	**Key Outcomes**
Li et al. (2021)	Rats with myocardial ischemia/reperfusion injury	100 mg/kg	Acute (pre-treatment and post-reperfusion)	Reduced infarct size and apoptosis via PI3K/Akt pathway
Zhao et al. (2022)	Mice treated with doxorubicin	Not explicitly stated	15 days (co-administered with doxorubicin)	Reduced myocardial apoptosis and oxidative stress via ECHS1 upregulation
Zhang et al. (2022)	LPS/cigarette smoke-induced COPD mice	40 and 80 mg/kg/day (oral)	6 weeks	Reduced airway inflammation, increased Aquaporin1 expression, decreased endothelial permeability
Shackebaei et al. (2024)	D-galactose-induced aged rats with I/R injury	40 or 100 mg/kg/day (oral)	8 weeks	Improved post-ischemic cardiac function, reduced TNF-α and oxidative stress
Muhammad et al. (2024)	Male and female Sprague–Dawley rats (alcohol + fructose gel)	50 mg/kg/day (oral, in gelatine)	10 weeks	Partially improved concentric remodeling; limited effect on diastolic dysfunction
Kaneriya et al. (2025)	LPS-treated Sprague-Dawley rats	Not clearly stated (therapeutic dose implied)	6 weeks	Improved endothelial function, NO bioavailability, reduced iNOS and MDA
Zhang et al. (2025)	ApoE-/- dyslipidemic mice	50, 100, or 200 mg/kg/day (oral)	4 weeks	Reduced cholesterol and oxidative stress, improved endothelial structure
Zhang et al. (2025)	ApoE—/— mice (atherosclerosis model)	Not explicitly stated	Not explicitly stated	Reduced atherosclerotic progression via PI3K-AKT/TLR4/NF-κB inhibition

Khamseekaew et al. (2025)	L-NAME-induced hypertensive rats	20 and 40 mg/kg/day (oral)	5 weeks	Improved cardiac remodeling and aortic dysfunction via RAS pathway inhibition
Zavodnik et al. (2025)	Streptozotocin-induced diabetic rats	40 mg/kg/day (oral)	4 weeks	Improved mitochondrial respiration and membrane potential, reduced oxidative stress

FIGURE S2: SYRCLE BASED Risk of Bias (2) Assessment for Animal Studies

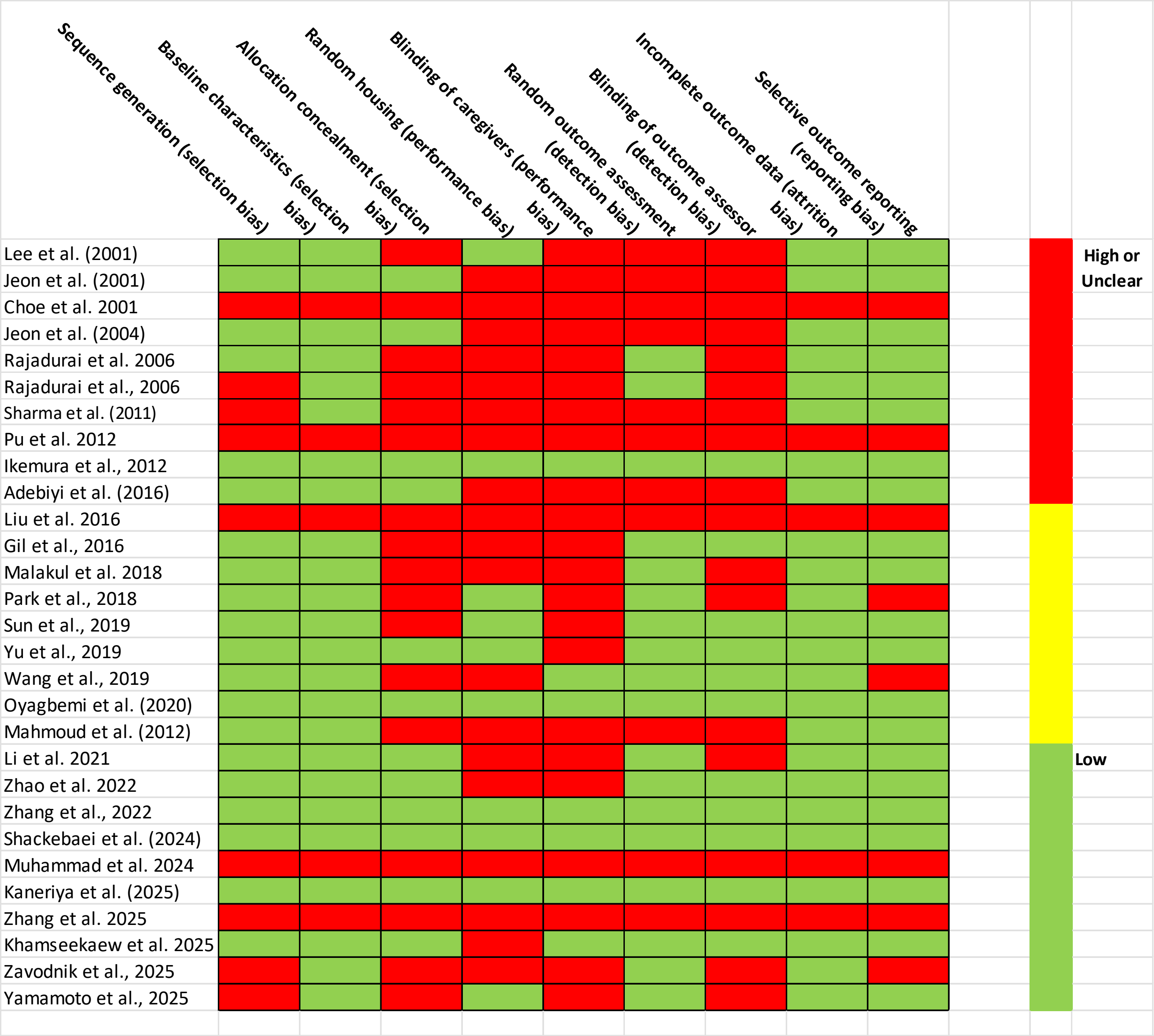

Supplement: Supplementary file 1 [file nutrients-17-02658-s001.zip › nutrients-3798333-supplementary.pdf]
